# Correction: The cost-effectiveness of controlling cervical cancer using a new 9-valent human papillomavirus vaccine among school-aged girls in Australia

**DOI:** 10.1371/journal.pone.0295688

**Published:** 2023-12-05

**Authors:** Rashidul Alam Mahumud, Khorshed Alam, Jeff Dunn, Jeff Gow

There is an error in the affiliations for the first author. Specifically, #3 was included in error and should not have been indicated. The correct affiliations are as follows:

Rashidul Alam Mahumud^1,2,3*^, Khorshed Alam^1,2^, Jeff Dunn^1,4,5^, Jeff Gow^1,2,6^**1** Health Economics and Policy Research, Centre for Health, Informatics and Economic Research, University of Southern Queensland, Toowoomba, Queensland, Australia, **2** School of Commerce, University of Southern Queensland, Toowoomba, QLD Australia, **3** Health and Epidemiology Research, Department of Statistics, University of Rajshahi, Rajshahi, Bangladesh, **4** Cancer Research Centre, Cancer Council Queensland, Fortitude Valley, QLD, Australia, **5** Prostate Cancer Foundation of Australia, St Leonards NSW, Australia, **6** School of Accounting, Economics and Finance, University of KwaZulu-Natal, Durban, South Africa

Additionally, there was an error in the corresponding author’s listed email addresses. Correspondence regarding this article should only be sent to the following email address: rashed.mahumud@usq.edu.au.

## References

[1] Mahumud RA, Alam K, Dunn J, Gow J (2019) The cost-effectiveness of controlling cervical cancer using a new 9-valent human papillomavirus vaccine among school-aged girls in Australia. PLoS ONE 14(10): e0223658. 10.1371/journal.pone.0223658 31596899 PMC6785120

