# Supplementary material for: The cost-effectiveness of controlling cervical cancer using a new 9-valent human papillomavirus vaccine among school-aged girls in Australia
Source: PLoS One. 2019 Oct 9;14(10):e0223658. doi: 10.1371/journal.pone.0223658 (PMC6785120; doi:10.1371/journal.pone.0223658)
Supplement: Attachment — Submitted filename: Response to Reviewers.docx [file pone.0223658.s002.docx]

**Response to the editor’s and reviewer’s comments**

Date: 7 September 2019

Dear Reviewer,

Thank you for giving us an opportunity to revise our manuscript entitled “*The cost-effectiveness of controlling cervical cancer using a new 9-valent human papillomavirus vaccine among school-aged girls in Australia*”. We found the reviewers’ comments/feedback very helpful in improving the manuscript and we have revised the manuscript accordingly. Please find attached the revised manuscript. We declare that all authors have no conflicts of interest. The manuscript has not been published in any other journal. Our point-by-point comments on the suggested revisions are below.

Best regards,

Rashidul Alam Mahumud (corresponding author)

PhD Candidate, MPH, MSc

On behalf of all of the co-authors

Health Economics Research,

Health Systems and Population Studies Division,

International Centre for Diarrhoeal Disease Research, Bangladesh (icddr,b),

Dhaka-1212, Bangladesh.

**Journal Requirements:**

**Comment 1.** When submitting your revision, we need you to address these additional requirements.

Please ensure that your manuscript meets PLOS ONE's style requirements, including those for file naming. The PLOS ONE style templates can be found at
<http://www.journals.plos.org/plosone/s/file?id=wjVg/PLOSOne_formatting_sample_main_body.pdf> and <http://www.journals.plos.org/plosone/s/file?id=ba62/PLOSOne_formatting_sample_title_authors_affiliations.pdf>

**Response:**

*This manuscript has been revised according to the journal style requirements (please see the revised manuscript).*

**Comment 2.** Please clearly list in your methods section or as supporting information, all the data sources and corresponding references used in this study. Additionally, if you collected any data that were not obtained from previously published research, provide details as to where these data are from and how they were collected.

***Response:***

*The supporting information and data have been described in the methods section. We have also described all data collection process and sources related to vaccine delivery costs and cervical cancer treatment costs including direct costs (e.g., medical and non-medical costs), indirect costs (e.g., productivity losses). Please see page 10 (lines 6-34) and page 11 (lines 1-8).*

**Comment 3.** We ask that you please use a more technically clear term to replace "sexual debut" as this not well defined in the manuscript and could be misinterpreted.

***Response:***

*We have checked and explained the technical words used in this manuscript. We have removed the word “sexual debut” from the revised manuscript. Please see page 6 (line-26).*

**Response to Reviewer #1 comments:**

**Comment:** I found the work interesting and very detailed. Overall it was quite comprehensive, evaluating many parameters that may affect cost-effectiveness studies. My only suggestion concerns the quality of the figures, I believe they could improve a little more.

***Response:*** *Authors express gratitude to the reviewer for their appreciation. The figures have been revised as per journal requirements. Please see the revised figures.*

**Response to Reviewer #2 comments:**

**Comment-1:**

The objective of this study was to examine the cost-effectiveness of adding a 9vHPV vaccine to the national immunization schedule in Australia across three different delivery strategies. The study is very interesting and important. The methods and results are described in detail. The conclusions are presented in an appropriate fashion and are supported by the data. The article is presented in an intelligible fashion.

***Response:*** *Authors express gratitude to the reviewer for their appreciation.*

**Comment-2**: I would like make some observations: In the objectives (page 5, line 26) the term “DALYs” is used by first time, please describe this acronym in this moment.

***Response:*** *The text has been revised now by describing the term DALY (i.e., disability adjusted-life years) as advised by the reviewer. Please see page 6 (lines 7-8).*

**Comment-3:** In the results (Page 12, line 11) the acronym “IECR” should be substituted by “ICER”?

***Response:*** *Authors are thankful to the reviewer for raising the issue. We have corrected the acronym (i.e., ICER). Please see page 12 (line-26).*
