# Supplementary material for: The cost-effectiveness of controlling cervical cancer using a new 9-valent human papillomavirus vaccine among school-aged girls in Australia
Source: PLoS One. 2019 Oct 9;14(10):e0223658. doi: 10.1371/journal.pone.0223658 (PMC6785120; doi:10.1371/journal.pone.0223658)
Supplement: Attachment — Submitted filename: Response to Reviewers.docx [file pone.0223658.s003.docx]

**Response to the editor’s and reviewer’s comments**

Date: 23 September 2019

Dear Reviewer,

Thank you for giving us an opportunity to revise our manuscript entitled “*The cost-effectiveness of controlling cervical cancer using a new 9-valent human papillomavirus vaccine among school-aged girls in Australia*”. We found the reviewers’ comments/feedback very helpful in improving the manuscript and we have revised the manuscript accordingly. Please find attached the revised manuscript. We declare that all authors have no conflicts of interest. The manuscript has not been published in any other journal. Our point-by-point comments on the suggested revisions are below.

Best regards,

Rashidul Alam Mahumud (corresponding author)

PhD Candidate, MPH, MSc

On behalf of all of the co-authors

Health Economics Research,

Health Systems and Population Studies Division,

International Centre for Diarrhoeal Disease Research, Bangladesh (icddr,b),

Dhaka-1212, Bangladesh.

**Response to Reviewer #1 comments:**

**Comment 1.** All comments have been addressed. After reviewing the work again, I noticed small issues that can be easily fixed:

**Response:** *We found the reviewers’ comments/feedback very helpful in improving the manuscript and we have revised the manuscript accordingly (please see the revised manuscript).*

**Comment 2.** On p. 2, line 7 and p. 3, line 23, please replace genotype by type, it is the most correct term today to refer to HPV types.

**Response:** *Thank you for your valuable concerns. The text has been corrected now accordingly (please see on page 2 (line-7) and page 3 (line-23)).*

**Comment 3.** On p. 5, line 12, replace the term nonavalent-HPV vaccine with the abbreviation (9vHPV) that has been previously used.

**Response:** *The term “nonavalent-HPV vaccine” has been replaced by “9vHPV” (please see on page 5, line-12).*

**Comment 4.** On p. 7, line 30, describe the term PBAC.

**Response:** *The text has been revised by describing the term PBAC as defined “Pharmaceutical Benefits Advisory Committee” (please see on page 7, lines-28-29).*

**Comment 5.** On p. 8, line 6 describe the term WTP.

**Response:** *The term WTP has been explained as “Willingness-to-pay” (please see page on 8, lines-4-5).*

**Comment 6.** On p. 8, line 23 describe the term NIP.

**Response:** *The term NIP has been described as “national immunisation program” (please see on page 8, line-21).*

**Comment 7.** On p. 16, line 20, separate the words "costper".

**Response:** *Corrected. Please see on page 16, line-19-20.*

**Comment 8.** On p. 18, line 5, separate the words "effectiveamong".
**Response:** *Corrected. Please see on page 18 (line 5).*

**Response to Reviewer #2 comments:**

**Comment-1:** All comments have been addressed

***Response:*** *Authors express gratitude to the reviewer for their appreciation.*
